# The association between ambient fine particulate matter and incident adenocarcinoma subtype of lung cancer

**DOI:** 10.1186/s12940-017-0268-7

**Published:** 2017-06-24

**Authors:** Lida Gharibvand, W. Lawrence Beeson, David Shavlik, Raymond Knutsen, Mark Ghamsary, Samuel Soret, Synnove F. Knutsen

**Affiliations:** 1 School of Allied Health Professions, Loma Linda University, Loma Linda, CA USA; 2 Center for Nutrition, Healthy Lifestyle, and Disease Prevention, School of Public Health, Loma Linda University, Loma Linda, CA USA; 3 Adventist Health Study-2, School of Public Health, Loma Linda University, Loma Linda, CA USA; 4 Center for Community Resilience, School of Public Health, Loma Linda University, Loma Linda, CA USA; 5 Loma Linda University School of Public Health, 24951 North Circle Drive, Nichol Hall 2005, Loma Linda, CA 92350 USA

**Keywords:** Air pollution, Lung adenocarcinoma, Lung cancer, Particulate matter, Adventists, Non-smokers, Non-melanoma skin cancer

## Abstract

**Background:**

Adenocarcinoma (AC) is the most common lung cancer among non-smokers, but few studies have assessed the effect of PM_2.5_ on AC among never smokers. The purpose of this study was to assess the association between ambient PM_2.5_ and incident lung AC in the Adventist Health and Smog Study-2 (AHSMOG-2), a cohort of 80,044 non-smokers (81% never smokers) followed for 7.5 years (597,177 person-years) (2002–2011).

**Methods:**

Incident lung AC was identified through linkage with U.S. state cancer registries. Ambient PM_2.5_ levels at subjects’ residences were estimated for the years 2000 and 2001, immediately prior to study start.

**Results:**

A total of 164 incident lung AC occurred during follow-up. Each 10 μg/m^3^ increment in PM_2.5_ was associated with an increase in the hazard rate of lung AC [HR = 1.31 (95% confidence interval (CI) 0.87–1.97)] in the single-pollutant model. Excluding those with prevalent non-melanoma skin cancer (NMSC) strengthened the association with lung AC (HR = 1.62 (95% CI, 1.11–2.36) for each 10 μg/m^3^ PM_2.5_ increment. Also, limiting the analyses to subjects who spent more than 1 h/day outdoors, increased the estimate (HR = 1.55, 95% CI: 1.05, 2.30).

**Conclusions:**

Increased risk of AC was observed for each 10 μg/m^3^ increment in ambient PM_2.5_ concentrations. The risk was higher among those without prevalent NMSC and those who spent more than 1 h/day outdoors.

## Background

According to the American Cancer Society (ACS), lung adenocarcinoma (AC) is the most common subtype of lung cancer (LC) in non-smokers and more likely to occur in females and younger people [1]. An estimated 224,390 new cases of LC overall and about 90,000 incident lung AC cases (approx. 40% of LC) are expected in the U.S. in 2016 [2]. The temporal increase of AC incidence is causing concern [3–9] as the annual age-adjusted incidence rates of lung AC increased by 2.8% for women and 1.3% for men from 2004 to 2009 [4]. An ecological U.S. study concluded that long-term exposure to nitrogen oxides (NO_x_) may play a major role in the increase of lung AC over the last 50 years [7]. However, to our knowledge, NO_2_ has never been reported as a carcinogen and the International Agency for Research on Cancer (IARC) states that there is a lack of evidence for NO_x_ as an independent cancer risk factor, supported by a recent meta-analysis [10] showing only an increase of 4% in LC incidence for each 10 μg/m^3^ increase in NO_2._

Ambient fine particulate matter (PM_2.5_), on the other hand, is associated with risk of overall lung cancer [11–18], but the relationship between PM_2.5_ and incident lung AC has been less studied [12, 13, 19].

In 2013, the International Agency for Research on Cancer (IARC) concluded that exposure to outdoor air pollution causes lung cancer and classified outdoor air pollution in general and particulate matter (PM) in particular, as a Group 1 carcinogen to humans [20]. This conclusion was based on findings from several studies, especially the recent results from the European Study of Cohorts for Air Pollution Effects (ESCAPE) [13] as well as a meta-analyses study [21].

We have recently reported a positive and relatively strong association between ambient fine particulate air pollution (PM_2.5_) and overall LC incidence in the Adventist Health and Smog Study-2 (AHSMOG-2), with HR’s ranging from 1.43 to 1.68, depending on model, for each 10 μg/m^3^ increment in PM_2.5_ [22]. In the present study, we have assessed the association between ambient PM_2.5_ and incident AC of the lung. We also studied the independent association between ambient O_3_ and LC incidence in a two-pollutant model with PM_2.5_ because of our previous findings of such association [23]. All study subjects were non-smokers, with 81% being never smokers, thus virtually eliminating the confounding effect of smoking.

## Methods

### Study population

**Fig. 1:**
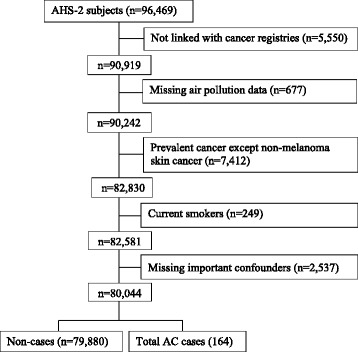
Study flowchart for adenocarcinoma (AC) subtype of lung cancer

The study population is the U.S. portion of the Adventist Health Study-2 (AHS-2), a large cohort study of about 96,000 subjects that has been described in detail elsewhere [24]. At enrollment, subjects completed a large 50-page questionnaire which can be viewed at www.adventisthealthstudy.org. Subjects were excluded from the current analysis if they were not linked with state cancer registries (Canadians and subjects living in Maine where we were not able to obtain permission to link with the state cancer registry) (*n* = 5550); had incomplete address information making it impossible to estimate residence specific air pollution concentrations (*n* = 677); reported prevalent cancers except non-melanoma skin cancer (*n* = 7412); were current smokers (*n* = 249) or had missing values on important covariates (*n* = 2537). These exclusions resulted in an analytic study population of 80,044 subjects (Fig. 1).

Written informed consent was obtained from all participants upon enrollment into the parent study (AHS-2) and this included subsequent analysis using de-identified data. The study was approved by the Loma Linda University Institutional Review Board.

### Outcome assessment

Incident cases were classified by the International Classification of Diseases for Oncology (ICD-O-3) codes C34.0-C34.9, with morphology codes including M-8046, 8140, 8200, 8250, 8252–8253, 8255, 8480–8481, and 8550 which were identified through computer-assisted record linkage with state cancer registries for the years 2002–2011. Subjects also completed several biennial mailed questionnaires regarding newly diagnosed cancers. If such self-reported cancers were not verified through the cancer registry linkage, medical records were obtained to verify such cases [24]. The outcome in this study was primary AC of the lung with histology classification of “adenocarcinoma”. We also included histology code “8046” even though this code is specified as non-small cell carcinoma. Other types of incident LC, e.g. non-AC LC (*n* = 94) were censored at the time of diagnosis. In addition to linkage with the various state cancer registries, subjects were also linked with the National Death Index (NDI) to ascertain death during follow-up.

### Estimation of ambient air pollution concentrations

Ambient air pollution data were obtained from the U.S. Environmental Protection Agency (EPA) Air Quality System (AQS) for the fixed time period from January 2000 through December 2001, the 2 years immediately prior to the start of the AHSMOG-2 study. Based on the EPA AQS data and Geographic Information Systems (GIS)-based inverse-distance-weighted (IDW) interpolations, multiple monthly pollution surfaces were created for PM_2.5_ and O_3_ across the U.S. using ArcGIS software [25]. To minimize errors, the IDW interpolation parameters were selected by assessing the goodness of fit of alternative model configurations through mean prediction error and root-mean-square error estimates. Only months with at least 75% valid data were included in the exposure estimates. Monthly exposure averages were based on daily PM_2.5_ and hourly O_3_ (24-h average). The GIS-derived monthly exposure averages were used to accumulate and assign monthly concentrations of ambient O_3_ and PM_2.5_ to the geocoded baseline residential street level address of the subjects.

### Study covariates

Covariates for the model were selected a priori based on published studies and suspected relationships and included gender, race, smoking status, years since subject quit smoking, average number of cigarettes per day during all smoking years and educational level. Additional candidate covariates included calendar time, alcohol consumption, family income, body mass index (BMI), physical activity, and marital status.

Three additional variables were identified a priori as potential confounders and/or effect modifiers: hours/day spent outdoors, years of pre-study residence length at enrollment address and moving distance from enrollment address during follow-up.

Since non-melanoma skin cancer (NMSC) has been found, in some studies, to increase the risk of non-skin cancers including lung cancer [26–29], we also decided a priori to do sensitivity analyses excluding those with NMSC at baseline.

### Statistical analysis

Baseline characteristics of cases and non-cases were compared using Chi-square test for categorical and Student t-test for continuous variables. Cox proportional hazards regression modeling, with attained age as the time variable with left truncation by age at study entry, was used for multivariable analyses. The Cox regression was augmented by adding the sandwich variance estimate [30] to adjust for correlated observations within each county. Participants were censored at time of diagnosis or, for non-cases, at the time of last linkage with the cancer registry or date of death, whichever came first.

Single- (PM_2.5_) and two-pollutant (PM_2.5_ and O_3_) analyses were conducted to assess the role of ambient PM_2.5_ on lung AC incidence. Pollutants were entered into the model as continuous variables and hazard ratios (HR) were calculated for an increment of 10 μg/m^3^ for PM_2.5_ and 10 ppb for average 24-h O_3_. The lowest increment for PM_2.5_ started with the lowest estimate of ambient air pollution registered in the study population.

The multivariable model was specified based on the pollutant(s) and the a priori selected covariables. Smoking was used as a nested covariate (smoke status + [smoke status × years since quit smoking] + [smoke status × years since quit smoking × cigarettes per day]). We dichotomized years since quit smoking (<20 and ≥20), and number of cigarettes per day (<8.5 and ≥8.5) based on the median levels. The additional candidate covariables (calendar time, alcohol consumption, family income, body mass index (BMI), physical activity, and marital status) were evaluated for inclusion in the model, but adding them did not change the main effect and they were therefore not included in the final model.

The three additional a priori potential effect modifiers (time spent outdoors, residence length and moving distance during follow-up) were then all added to Model 1 as covariates. When testing for effect modification by smoking, it was important to compare our findings with that of others. Therefore, the nested smoking variable was replaced by a dichotomized smoking variable (quit smoking <10 years ago and quit smoking ≥10 years ago combined with never smokers) which previously has been used by the Nurses’ Health Study (NHS) when testing for effect modification of air pollution and lung cancer [12]. Additionally, a subgroup analyses was also performed to separately assess the risk estimates for PM_2.5_ on lung AC in past and never smokers.

The Cox HR proportionality assumption was evaluated using Schönfeld residuals, log (−log) plots, and time (attained-age) product terms and no departure from proportionality was evident. Furthermore, using multiple linear regressions, no multicollinearity was evident between covariates. Assessment of Schönfeld residuals did not show important influential data points. The linearity assumptions for the exposure variables were tested and were not in violation of the proportional hazards assumption. All statistical analyses were performed using SAS 9.4 (SAS Institute, Inc. Cary, NC).

## Results

### Study population description

One hundred and sixty four histologically confirmed lung AC cases (27.5 cases per 100,000 person-years) were diagnosed during a median follow-up of 7.5 years (597,177 person-years). Cases tended to be older, past smokers, have lower educational levels, spend more time outside, were more likely to have used alcohol and had lived longer at their enrollment address. They were also more likely to have quit smoking more recently, to have been heavier smokers; and to report prevalent non-melanoma skin cancer (Table 1). During follow-up, 20% of the subjects (*n* = 15,998) moved more than 30 km away from their baseline place of residence while 61% (*n* = 48,960) did not move during follow-up.

**Table 1:** Selected characteristics of the study population at baseline

Characteristics	Non-Cases (*n* = 79,880)	Cases (*n* = 164)	P-Valve
Age	57.02 ± 14.22	68.07 ± 10.85	<.001
PM_2.5_ (μg/m^3^)	12.88 ± 3.72	13.11 ± 3.98	<.001
Ozone 24 h (ppb)	26.88 ± 3.89	27.07 ± 4.22	<.001
Gender			0.730
Females	52,067 (65.2%)	109 (66.5%)	
Males	27,813 (34.8%)	55 (33.5%)	
Smoking Status			<.001
Never Smokers	64,817 (81.1%)	89 (54.3%)	
Past smokers	15,063 (18.9%)	75 (45.7%)	
Race			0.599
Blacks	57,461 (71.9%)	121 (73.8%)	
Non-Blacks	22,419 (28.1%)	43 (26.2%)	
Education			<.001
High school or less	21,820 (27.3%)	77 (47.0%)	
Trade school/ associate degree/ some college	27,112 (33.9%)	52 (31.7%)	
Bachelor degree+	30,948 (38.7%)	35 (21.3%)	
Family Income			<.001
Less than $31,000	41,247(51.6%)	110 (67.1%)	
$31,000–$75,000	23,534 (29.5%)	41 (25.0%)	
$75,000 or more	15,099 (18.9%)	13 (7.9%)	
Body Mass Index (kg/m^2^)^a^			0.280
Less than 25	30,411 (39.3%)	54 (34.4%)	
25–29.99	27,034 (34.9%)	64 (40.8%)	
30 or more	19,993 (25.8%)	39 (24.8%)	
Physical Activity			0.290
Low	31,401 (39.3%)	74 (45.1%)	
Medium	33,472 (41.9%)	64 (39.0%)	
High	15,007 (18.8%)	26 (15.9%)	
Hours Per Day Spent Outdoors			0.003
Less than 1 h/day	19,508 (24.4%)	39 (23.8%)	
1–3.5 h/day	45,156 (56.5%)	77 (47.0%)	
More than 3.5 h/day	15,216 (19.1%)	48 (29.3%)	
Alcohol Status^a^			<.001
Never	46,935 (59.1%)	73 (44.8%)	
Ever	32,537 (40.9%)	90 (55.2%)	
Residence Length^b^			<.001
Less than 5 years	19,924 (25.0%)	29 (17.7%)	
5 ≤ years <12	20,583 (25.8%)	32 (19.5%)	
12 ≤ years <24	19,713 (24.7%)	43 (26.2%)	
More than 24 years	19,660 (24.6%)	60 (36.6%)	
Moving Distance^c^			0.649
0 KM	48,865 (61.2%)	95 (57.9%)	
0 < KM ≤30	15,054 (18.9%)	32 (19.5%)	
More than 30 KM	15,961 (20.0%)	37 (22.6%)	
Years Since Quit Smoking (7 levels)			<.001
Never smokers	64,817 (81.1%)	89 (54.3%)	
Quit ≥30	4738 (5.9%)	19 (11.6%)	
Quit 20–29.9 years	3601 (4.5%)	15 (9.2%)	
Quit 10–19.9 years	3170 (4.0%)	17 (10.4%)	
Quit 5–9.9 years	1394 (1.8%)	7 (4.3%)	
Quit 1–4.9 years	1199 (1.5%)	8 (4.9%)	
Quit <1 year	961 (1.2%)	9 (5.5%)	
Average Number of Cigarettes Per Day			<.001
None	64,906 (81.1%)	89 (54.3%)	
Less than 8.5	7677 (9.6%)	27 (16.5%)	
Greater or equal to 8.5	7461 (9.3%)	48 (29.3%)	
Non-Melanoma Skin Cancer			<.001
No	74,529 (93.3%)	142 (86.6%)	
Yes	5351 (6.7%)	22 (13.4%)	

Values are presented as mean ± SD or no. (%)

^a^Some columns do not add to 100% because of missing data

^b^Years of Pre-Study Residence within 10 miles of Enrollment Address

^c^Distance of Moving During Follow-up of Initial Place of Residence

**Fig. 2:**
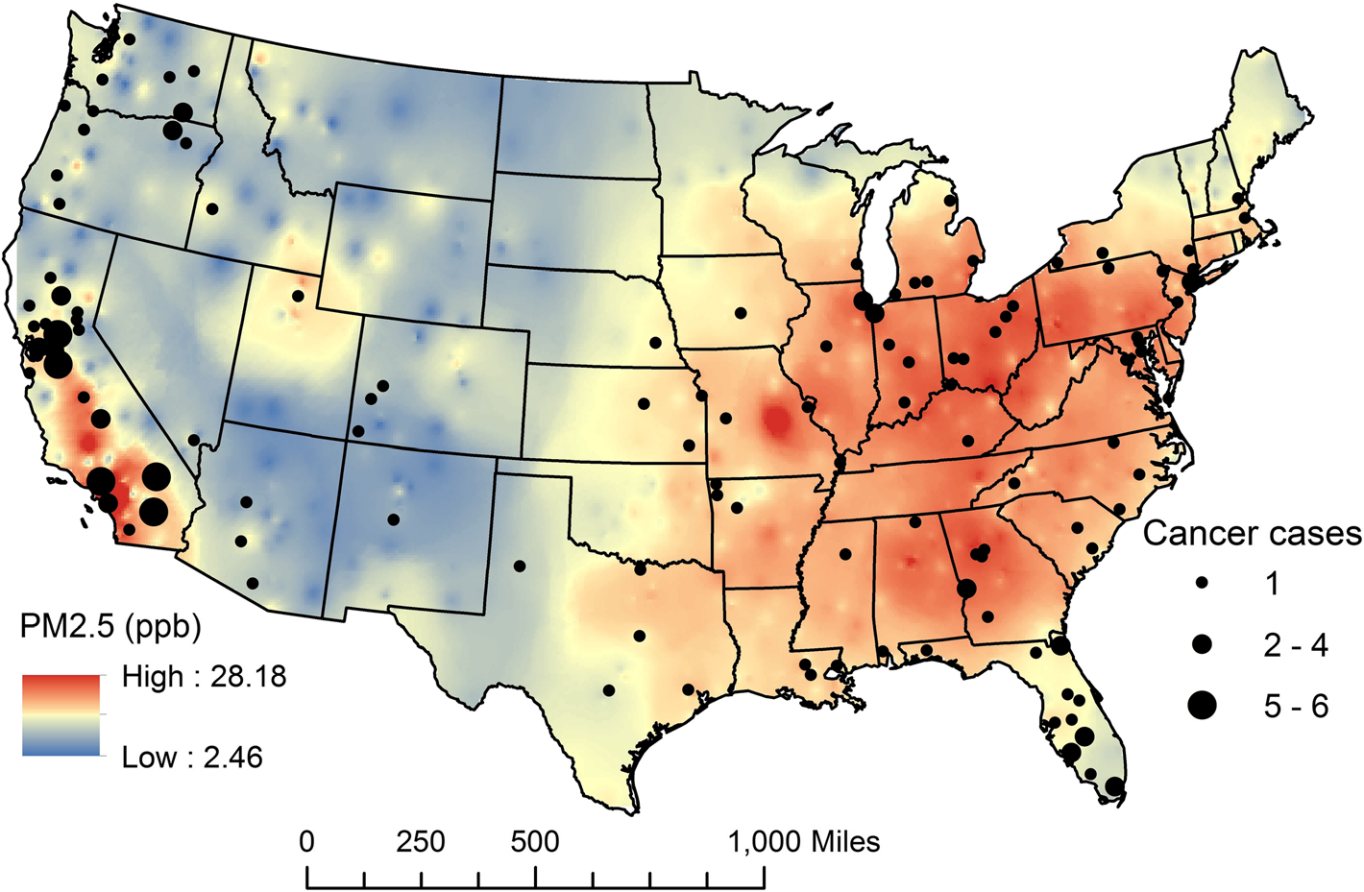
Lung adenocarcinoma cases (*n* = 164) overlaid on ambient PM_2.5_ surface (2000–2001 average)

Non-cases consisted mainly of never smokers, 81% (*n* = 64,817), with the remaining 19% (*n* = 15,063) being past smokers. In contrast, among the 164 lung AC cases, 89 (54%) were never smokers while 75 (46%) were past smokers (Table 1). The average 24-h ozone concentrations for subjects without and with lung AC were 26.9 (range: 14.1–46.7) and 27.1 (range: 18.3–41.9) ppb, respectively, while mean PM_2.5_ concentrations were 12.9 (range: 4.1–26.5) and 13.1 (range: 5.3–22.4) μg/m^3^, respectively. Figure 2 shows adenocarcinoma subtype of lung cancer cases (*n* = 164) overlaid on a PM_2.5_ monthly exposure surface (average of years 2000–2001) generated through GIS-based inverse-distance-weighted (IDW) interpolation of monitored data across the U.S. EPA AQS network.

Exposure assignments were based on actual subject’s residence location. However, for confidentiality reason, the actual location of cases on the U.S. map was masked by placing them at the geographic center of the corresponding residential county.

### PM_2.5_ and O_3_ effects

A positive association was found between ambient PM_2.5_ and incident lung AC in the two-pollutant sandwich variance estimated model with O_3_ [HR = 1.31 (95% CI: 0.92, 1.86)] for each 10 μg/m^3^ increment in PM_2.5_. This estimate was virtually identical to the estimates from the models without the sandwich variance estimate. Adding the three additional potential confounders/effect modifiers as covariates, did not change the main effect [HR = 1.32 (95% CI: 0.93, 1.89)] (Table 2, Model 2).

**Table 2:** Multivariable-adjusted HRs for incident lung adenocarcinoma per 10-μg/m^3^ increment in mean monthly ambient PM_2.5_: single- and two-pollutant models. 79,880 AHSMOG-2 subjects (LC cases = 164)

	Pollutant	cases	Single Pollutant HR (95% CI)	Two Pollutant^a^ HR (95% CI)	Two Pollutant^ab^ HR (95% CI)
Model 1	PM_2.5_	164	1.31 (0.87, 1.97)	1.31 (0.87, 1.97)	1.31 (0.92, 1.86)
	O_3_			1.01 (0.68, 1.50)	1.01 (0.69, 1.47)
Model 2	PM_2.5_	164	1.32 (0.88, 1.99)	1.32 (0.88, 2.00)	1.32 (0.93, 1.89)
	O_3_			1.02 (0.68, 1.51)	1.02 (0.70, 1.47)
Model 3					
Outdoors <1	PM_2.5_	39	0.75 (0.32, 1.75)	0.75 (0.32, 1.75)	0.75 (0.35, 1.61)
Outdoors ≥1	PM_2.5_	125	1.55 (0.98, 2.46)	1.55 (0.98, 2.46)	1.55 (1.05, 2.30)
Sensitivity Analysis					
Model 4					
Excluding NMSC	PM_2.5_	142	1.62 (1.04, 2.50)	1.62 (1.04, 2.51)	1.62 (1.11, 2.36)

Model 1– Adjusted for gender, educational level, race, and nested covariates: smoking status, years since quit smoking, and average number of cigarettes per day

Model 2–Model 1 + outdoors, residence length, moving distance, NMSC

Model 3–Model 1+ outdoors + PM_2.5_* outdoors (2 levels of outdoors: <1 & ≥1 h/day)

Model 4–Model 1, but excluding prevalent NMSC^a^Model [1–4] – adjusted for O_3_ with increments of 10 ppb

^a^Model [1–4] – adjusted for O_3_ with increments of 10 ppb

^b^Model [1–4] – with Sandwich variance estimate

No independent association with LC was found for O_3_ in the two-pollutant multivariable model, HR = 1.01 (95% CI: 0.68, 1.50) for each 10 ppb increment in 24-h levels (Table 2, Model 1).

### Effect modifications

Among the identified three a priori potential effect modifiers (time spent outdoors, residence length and moving distance) only time spent outdoors marginally modified the association (p_interaction_ = 0.09). Among those spending more than 1 h/day outside, the HR increased to 1.55 (95% CI: 1.05, 2.30) whereas no effect of ambient PM_2.5_ was observed for those spending less than 1 h/day outdoors (Table 2, Model 3).

There was no effect modification by smoking when stratifying on years since quitting smoking (p_interaction_ = 0.99) nor when stratifying on past vs. never smokers (p_interaction_ = 0.42). There was, however, slightly stronger estimates for the effect of PM_2.5_ on lung AC among past smokers in the two-pollutant models with O_3,_ [HR = 1.55 (95% CI: 0.84, 2.89)] vs never smokers [HR = 1.13 (95% CI: 0.66, 1.93)], but the 95% CI were wide.

### Sensitivity analysis

A sensitivity analysis was conducted by excluding 5373 subjects with prevalent NMSC from the final multivariable model. When limiting the analyses to those with no prevalent NMSC, the HR for lung AC, associated with PM_2.5_, was strengthened [HR = 1.62 (95% CI: 1.11, 2.36)] (Table 2, Model 4).

## Discussion

Our finding of a 31% increase in incident lung AC associated with each 10 μg/m^3^ increment in ambient PM_2.5_ concentration is in line with findings of the NHS (33% increase) [12] and stronger than that reported by the Netherlands Study (25% increase) [19]. The ESCAPE meta-analytic study found an even stronger effect (55% increase) per 5 μg/m^3^ incremental increase in PM_2.5_ [13].

The NHS, the largest study of incident LC among US women to date, and with a follow-up of 16 years, examined the relation of lung AC incidence with ambient PM and residential distance to roadways. Similar to our findings, an increase in lung AC was observed for each 10 μg/m^3^ increment of PM_2.5_ (HR = 1.33; 95% CI: 0.92–1.93). The association was even stronger when limiting the analyses to never smokers or smokers who quit ≥10 years ago (HR = 1.66; 95% CI: 0.81–3.42) [12] which is different from our data where the estimates was somewhat higher among the past smokers, although with wide confidence intervals due to small numbers. Also, the reference groups were different in that it included current smokers in the NHS, but non-smoking recent (<10 years ago) quitters in our analyses.

An extended follow-up of the prospective Netherlands Cohort Study on Diet and Cancer (NLCS) investigated the association of air pollution with incident lung cancer by histological subtypes and evaluated the impact of air pollution exposure measurement error on the risk of incident lung cancer [19]. After adjusting for measurement error using regression calibration, the HR for incident lung AC increased from 1.12 (95% CI: 0.74–1.70) in the multivariable model to 1.25 (95% CI 0.54–2.89) per 10 μg/m^3^ increment in PM_2.5_. The authors conclude that their measurement error adjustments “provides a sense of the level of underestimation in studies that are unable to perform this correction for measurement error bias”. Unfortunately, we were not able to do measurement error adjustment, but this latest report from the NLCS suggests that our findings may be underestimates of the true association between ambient PM_2.5_ and incident lung AC.

Compared to our study with 164 cases, the larger European Study of Cohorts for Air Pollution Effects (ESCAPE), with 727 AC cases and 12.8 years of follow-up, reported a stronger HR of 1.55 (95% CI: 1.05–2.29) for each 5 μg/m^3^ increment in PM_2.5_ [13]. In another meta-analysis of 17 cohorts, Hamra et al. [21] reported a meta-estimate HR of lung AC of 1.40 (95% CI: 1.07–1.83) for each 10 μg/m^3^ increase in PM_2.5_.

A Canadian case control study [11] reported increased odds of incident lung AC (OR = 1.27 (95% CI: 0.84–1.90) for each 10 μg/m^3^ increase in PM_2.5_ and OR = 1.17 (95% CI: 1.01–1.35) for each 10 ppb increase in NO_2_. For each 10 ppb increase in O_3_ the OR was 1.04 (95% CI: 0.74–1.44), very similar to our findings.

Although not a study of PM_2.5_ and lung AC per se, another case control study in Canada investigated the associations between incident lung cancer subtypes and occupational exposure to diesel and gasoline engine emissions, both known markers of PM_2.5_ pollution. At least 10 years occupational exposure to diesel engine emission was not associated with lung AC and 10 years exposure to gasoline engine emissions was only weakly associated with lung AC with adjusted ORs of 0.94 (95% CI: 0.72–1.25) and 1.10 (95% CI: 0.83–1.44), respectively. The potential for exposure misclassification and the inability to take into account non-occupational exposure to gasoline in particular, in the authors’ view, implied that the risks were likely to be underestimated [31].

Nitrogen oxides (NO_x_) are also considered markers of PM_2.5_. A Danish study, using outcome data from three prospective cohort studies, however, found no clear association between ambient NO_x_ and incidence of lung AC [32].

We found no association between lung cancer and ozone levels, even after controlling for PM_2.5_ in two-pollutant models. Only a few studies have assessed the relationship of ozone with LC and most have found no association [11, 33].

In contrast, in the previous and smaller AHSMOG-1 study where a different ozone metric was used, we found an increased risk of LC among males [23] with a relative risk RR of 3.56 (95% CI: 1.35, 9.42) for every 556 h/year that the males experienced O_3_ levels of 100 ppb or higher. There was no association between ambient O_3_ levels and incident LC among females. In that study, males, as compared to females, spent considerably more time outdoors, especially in the summer, and reported twice as much vigorous exercise outdoors in the summer, when O_3_ was higher. Further, these AHSMOG-1 findings were related to exceedance frequencies of O_3_ (i.e. hours/year that an air pollutant exceeded a specific concentration) which are a different O_3_ metric than the 24-h average used in the present study.

We found that the HR for lung AC associated with ambient PM_2.5_ was strengthened when excluding prevalent NMSC. As far as we can tell, among 15 studies assessing the relationship between ambient air pollution and lung AC, none of them specifically say they have excluded NMSC and only one of 35 studies which assessed the association between ambient NOx and total LC has specifically mentioned excluding NMSC [34]. Several studies [26–29], but not all [35], have reported increased risk of non-cutaneous cancer among subjects with prevalent NMSC. Silverberg and Ratner found that both NMSC and melanoma were associated with increased odds of non-cutaneous malignancies including lung cancer [26]. Rees, et al. reported increased risk of lung cancer among subjects with prevalent basal cell cancer (BCC) HR = 1.14 (95% CI: 0.68–1.90), but no increased risk after squamous cell skin carcinomas (SQCC) [27]. A prospective study in Switzerland found that prevalent SQCC was associated with an excess risk of lung cancer standardized incidence ratio (SIR) [1.3 (95% CI: 1.0–1.6)], and other non-cutaneous cancers [28]. A study in Finland followed BCC patients for 9 years and found increased risk of several non-cutaneous sites, including lung/trachea cancer SIR = 1.12 (95% CI:1.06–1.17) [29]. Levi, et al., however, concluded that subjects diagnosed with BCC do not have a generalized excess risk of non-cutaneous neoplasms, with the exception of non-Hodgkin’s lymphoma and cancers of the lip and salivary glands [35]. To our knowledge, the findings of a stronger association between ambient fine particulates and risk of lung AC when excluding prevalent NMSC have not been reported previously. Replicating these findings in other large cohort studies is important. If confirmed, possible biologic mechanisms are unclear and speculative at this time. Serum vitamin D levels have been found to be inversely related to LC incidence [36, 37] and diets high in fruits and vegetables have also been reported as protective for LC development [38–40]. However, to our knowledge, no previous study has assessed possible effect modifications of NMSC on the association between smoking or air pollution on risk of LC. It is possible that subjects with prevalent NMSC modify their lifestyle by spending less time outdoors, at least during daylight hours when sunlight is strong and air pollution is higher, eating a more healthy diet and taking vitamin D supplements. But these possible relationships, as well as other possible biologic mechanisms, need to be explored further. Our findings, however, do raise the question of whether exclusion of prevalent NMSC should be routine when assessing risk of LC associated with ambient air pollution in longitudinal studies.

### Strengths and limitations of the study

There are several strengths of this study. This is a health conscious non-smoking population where almost 81% have never smoked and where 55% of past smokers quit more than 20 years ago. The proportion who currently use alcohol is low and the actual use among the current drinkers is very low. Thus there is minimal confounding by smoking or alcohol. Another strength is that we have ambient air pollution estimates at the residence street level which makes individual ambient air pollution estimates more valid. Since Black subjects have been under-represented in most cohort studies, the relatively large proportion (28%) of Black subjects in this study is important. The validity of the study is strengthened by our ability to adjust for time spent outdoors, length of residence at enrollment address and moving history during follow-up as well as the fact that we were able to do linkage to state cancer registries to obtain lung cancer incidence.

One potential limitation is that we did not specifically ask about environmental tobacco smoke (ETS) in our baseline questionnaire. However, all subjects in this cohort are Adventists and typically live in households with other Adventists and so we believe the prevalence of ETS is very low especially because smoking in the workplace was unlikely in the early 2000’s given legislation in the various states [41]. We only assessed ambient air pollution during the 2 years immediately prior to study start and this could potentially attenuate our results. Another possible and unmeasured source of ambient PM_2.5_ is tailpipe emissions which is known to have higher concentrations of PM_2.5_ than the typical residential areas [42, 43]. We have no information on how many hours subjects spend in motor vehicle traffic, but such information at the individual level could potentially modify the observed associations we have reported. Lastly, the residence-specific air pollution estimates were based on air quality monitoring stations, and not on personal monitoring, and this may result in unknown amounts of misclassification. But such misclassification is most likely non-differential and would tend to bias results towards the null.

## Conclusions

In summary, this study showed a 31% increase in incident lung AC associated with incremental increase in ambient PM_2.5_ concentrations among non-smokers. Our findings are in line with other cohort studies and also support the conclusions of IARC in classifying outdoor air pollution and PM_2.5_ as carcinogenic. The potential impact of different chemical compositions of PM_2.5_ should be evaluated in future studies. The observed interaction with time spent outdoors is important and underscores the importance of legislation to control ambient levels of particulate air pollution. Our findings that the association between ambient air pollution and AC of the lung is strengthened when excluding prevalent NMSC has, to our knowledge, not been reported before and needs further study in other large cohort studies. If replicated, this finding could result in altered practice regarding exclusion of prevalent NMSC when assessing incident non-cutaneous cancers, especially those related to air pollution. Our findings are important for the public as they make informed choices about their lifestyle and place of residence.

## Abbreviations

AC: Adenocarcinoma
AHS-2: Adventist Health Study-2
AHSMOG: Adventist Health Study on the Health Effects of Smog
AQS: Air Quality System
BCC: Basal Cell Cancer
BMI: Body Mass Index
CI: Confidence Interval
EPA: Environmental Protection Agency
ESCAPE: European Study of Cohorts for Air Pollution Effects
ETS: Environmental Tobacco Smoke
GIS: Geographic Information Systems
HR: Hazard Ratio
ICD-O-3: International Classification of Diseases for Oncology
IDW: Inverse-Distance-Weighted
IRB: Institutional Review Board
LC: Lung Cancer
Log: Logarithm
NDI: National Death Index
NLCS: Netherlands Cohort Study
NMSC: Non-Melanoma Skin Cancer
NOx: Nitrogen Oxides
O_3_: Ozone
OR: Odds Ratio
PM_2.5_: Particulate Matter with Aerodynamic Diameter Smaller than 2.5 μm
RR: Relative Risk
SAS: SAS Institute - Cary, NC, USA
SIR: Standardized Incidence Ratio
SQCC: Squamous Cell Cancer
